# Current clinical practices in pediatric audiology among audiologists in India

**DOI:** 10.1371/journal.pone.0355049

**Published:** 2026-07-30

**Authors:** Twinkle Lijo Kirianthan, Dhanshree R. Gunjawate, Rohit Ravi

**Affiliations:** Department of Audiology and Speech-Language Pathology, Kasturba Medical College Mangalore, Manipal Academy of Higher Education, Manipal, India; LSU Health Shreveport, UNITED STATES OF AMERICA

## Abstract

**Objective:**

Evidence-based practice is central to audiology; however, adherence to best-practice guidelines varies, particularly in pediatric settings. This study explored current pediatric audiology practices among audiologists in India.

**Methods:**

A cross-sectional survey was conducted using an adapted version of the American Board of Audiology’s Pediatric Audiology Clinical Practice Analysis Survey modified for the Indian context. The survey was distributed electronically via email using contact information available with Rehabilitation Council of India (RCI). Eligible participants held at least a bachelor’s degree in Audiology and Speech-Language Pathology and had a minimum of one year of clinical experience. Participation was voluntary, informed consent was obtained, and confidentiality of responses was ensured. Descriptive statistical analyses were performed. Scale content validity index was calculated to assess content validity of the questionnaire.

**Results:**

A total of 130 audiologists participated, representing a predominantly early-career workforce (mean experience = 4.92 ± 5.41 years). Core pediatric audiology procedures were widely practiced. Newborn hearing screening was considered very important by 66.2% of participants, while 35.4% performed it several times daily. Tympanometry was rated very important by 61.5%, with 40% performing it several times daily. Counselling-related practices were strongly emphasized, with 68.5% rating explanation of test results as very important and 48.5% performing it several times daily. In contrast, advanced procedures demonstrated lower utilization. Cortical evoked potentials were not performed by 35.4% of participants, vestibular assessments by 43.1%, cochlear implant programming by 45.4%, and bone conduction device management by 57.7%.

**Conclusions:**

Indian audiologists consistently provide essential pediatric audiology services; however, gaps persist in specialized diagnostics and rehabilitation. Enhancing infrastructure, professional training, and adherence to evidence-based guidelines is necessary to strengthen pediatric audiological care in India.

## Introduction

Hearing loss remains a major global public health concern, affecting more than 1.5 billion individuals worldwide, with approximately 430 million requiring rehabilitation services [1]. The associated economic burden exceeds $981 billion globally [2]. In India, the high prevalence of childhood hearing loss underscores the importance of early identification and intervention [3,4]. However, access to pediatric audiology services remains limited due to shortages in workforce, infrastructure, referral systems, and specialized facilities [5,6]. Evidence-based practice (EBP) is a systematic approach that integrates clinical expertise with the best available research evidence while considering the values and preferences of patients and their families [7]. It involves formulating clinical questions, critically appraising relevant evidence, and applying findings to support patient-centred decision-making. Higher levels of evidence, such as randomized controlled trials, are generally regarded as more reliable and contribute to improved patient outcomes and greater healthcare efficiency [8]. In audiology, EBP has increasingly guided clinical assessment, intervention, and rehabilitation practices since the mid-1990s [9]. Since 2005, the American Speech-Language-Hearing Association (ASHA) has emphasized integrating research evidence with clinical expertise and family preferences, highlighting the clinician’s role in critically evaluating evidence and incorporating it into practice.

The development of evidence-based clinical guidelines requires clearly defined clinical questions, systematic literature review, and critical appraisal of evidence by multidisciplinary experts to ensure rigorous and unbiased recommendations [10]. However, in audiology, available research is often limited in quality or applicability, which may restrict the implementation of evidence-based recommendations in clinical settings [11]. Consequently, variability in clinical practice continues to exist despite established best-practice guidelines [12].

In pediatric audiology, evidence-based clinical practice is particularly important because early identification and timely intervention are critical for optimal speech, language, and developmental outcomes. Hearing screening programs conducted in newborn, preschool, and school settings are effective in identifying congenital, progressive, or late-onset hearing loss that might otherwise remain undetected [3,13]. Audiologists play a central role in the management of childhood hearing loss through age-appropriate assessment, counselling, referral, and ongoing follow-up services [14,15]. EBP has significantly influenced pediatric audiology practices, including assessment protocols, intervention strategies, and family counselling approaches [16,17].

Despite the availability of pediatric audiology guidelines, inconsistencies in implementation remain evident across clinical settings, particularly in counselling, assessment, and rehabilitation practices. Counselling forms an essential component of pediatric audiology and includes both informational counselling related to communication options, hearing technology, educational planning, and audiogram interpretation and adjustment counselling that supports families in coping with the emotional impact of childhood hearing loss [18]. The quality and clarity of counselling provided to families can directly influence timely decision-making and access to intervention services [15]. In addition, quality improvement frameworks emphasize continuous evaluation of clinical structures, processes, and outcomes to enhance the effectiveness and efficiency of audiology services [16].

Empirical evidence suggests considerable variability in pediatric audiology practices across countries. In India, Easwar et al. [19] provided an overview of audiological practice and reported the use of both behavioural and objective assessment methods by audiologists. However, their survey focused on general audiology practice rather than pediatric audiology specifically and did not comprehensively evaluate pediatric assessment, rehabilitation, counselling, case management, or professional practice. Furthermore, the study was conducted more than a decade ago, prior to several advances in pediatric hearing healthcare, including wider implementation of newborn hearing screening, updated evidence-based clinical guidelines, and developments in hearing technologies. Similarly, a North American survey demonstrated variation in pediatric audiology practice despite clinicians’ awareness of evidence-based recommendations [20]. More recently, Goyal et al. [21] examined rehabilitation practices for individuals with minimal-to-mild hearing loss and highlighted the need for clearer clinical guidelines and stronger rehabilitation support systems in India. However, to our knowledge, no recent study has systematically examined the full spectrum of contemporary pediatric audiology practices among audiologists in India using a pediatric-specific practice survey. Therefore, the present study aimed to explore current pediatric audiology practices in the Indian context.

## Method

The study was initiated after the approval of the Institutional Ethics Committee of Kasturba Medical College, Mangalore (IECKMCMLR02/ 2025/ 55). A cross-sectional web-based survey was conducted during the period from March 2025 to March 2026.

### Participants

Audiologists practicing in India who had at least one year of clinical experience and possessed a bachelor’s degree in Audiology and Speech-Language Pathology, a master’s degree in Audiology, or a PhD were included in the study. Audiologists practicing outside India, as well as those currently pursuing a master’s degree or PhD, were excluded from participation. Audiologists currently pursuing a master’s degree or PhD were excluded because their clinical responsibilities are often supervised and may not accurately represent independent clinical practice. Sample size was calculated using the formula n = (z² × p × q)/ e², with z = 1.96 (95% confidence level), p = 0.82 and q = 0.18 [22], and a margin of error (e) of 7%, yielding a required sample size of 116. The audiologists fulfilling the inclusion and exclusion criteria were included regardless of their pediatric caseload. pediatric services in India are typically provided by audiologists with diverse caseloads, rather than by formally designated pediatric specialists. Thus, a broad inclusion criterion was selected to accurately represent the contemporary clinical practices.

### Questionnaire details

Permission to adapt the Pediatric Audiology Clinical Practice Analysis Survey developed by the American Board of Audiology [23] was obtained prior to the study. The questionnaire was modified to suit the Indian clinical context and subsequently subjected to content validation by five experienced audiologists with more than 10 years of clinical experience. Experts evaluated the relevance of each item using a four-point rating scale (1 = relevant, 2 = somewhat relevant, 3 = quite relevant, and 4 = irrelevant) based on the criteria proposed by Davis [24]. Only items rated as relevant or somewhat relevant were retained in the final questionnaire. The Scale-Level Content Validity Index (S-CVI) was calculated following the method described by Polit and Beck [25], yielding a score of 0.86, indicating excellent content validity.

The final questionnaire, administered in English, comprised five domains: demographic details; assessment and diagnosis of auditory and vestibular disorders; habilitation and rehabilitation; case management and counselling; and professional and regulatory needs. The demographic section obtained information regarding age, gender, educational qualifications, years of clinical experience, primary workplace, and pediatric caseload. The remaining sections explored the perceived importance and frequency of various pediatric audiology practices, including diagnostic procedures, electrophysiological and vestibular assessments, rehabilitation services, counselling practices, case management activities, adherence to professional guidelines, infection control, and equipment calibration practices.

### Data collection

The questionnaire was created using Google Forms and included a study information sheet followed by informed consent form. Only participants who provided consent could access the questionnaire. Audiologists practicing in India based on the predefined inclusion and exclusion criteria were contacted. Participant contact details were obtained from the Rehabilitation Council of India (RCI) database, and the survey link was distributed through registered emails. The invitation email was sent once, followed by two reminder emails at three-week intervals. Participation was voluntary, and completion of the questionnaire required approximately 12–15 minutes. Responses were securely stored on Google Drive and were accessible only to the investigators to maintain confidentiality.

Descriptive statistical analyses were performed using Jamovi version 2.6. [26]. Continuous variables were summarized using mean and standard deviation, while categorical variables were presented as frequencies and percentages.

## Results

### Participant characteristics

A total of 130 audiologists participated, ranging from 22 to 49 years (mean = 28.36 ± 5.43 years) in age, with 60% (n = 74) female and 43% (n = 56) male. Professional experience ranged from 1 to 26 years (mean = 4.92 ± 5.41 years), indicating a predominantly early-career workforce. Majority held a master’s degree (n = 65, 50.0%), followed by a bachelor’s degree (n = 55, 42.3%) and only 7.7% (n = 10) had a doctoral degree (PhD). Regarding the primary practice setting, the largest proportion of participants worked in private clinics (n = 56, 43.1%), followed by universities/institutions (n = 39, 30.0%) and hospitals (n = 35, 26.9%). With respect to pediatric caseload, 30.8% of audiologists reported managing fewer than 30 pediatric clients. Additionally, 21.5% indicated that pediatric cases constituted 30–49% of their caseload, 31.5% reported 50–69%, 10.8% reported 70–89%, and only 5.4% managed predominantly pediatric caseloads (90–100%). These findings suggest that although the sample largely comprised young professionals, relatively few were extensively engaged in pediatric clinical practice.

### Assessment and diagnosis of auditory and vestibular disorders

**Fig 1:**
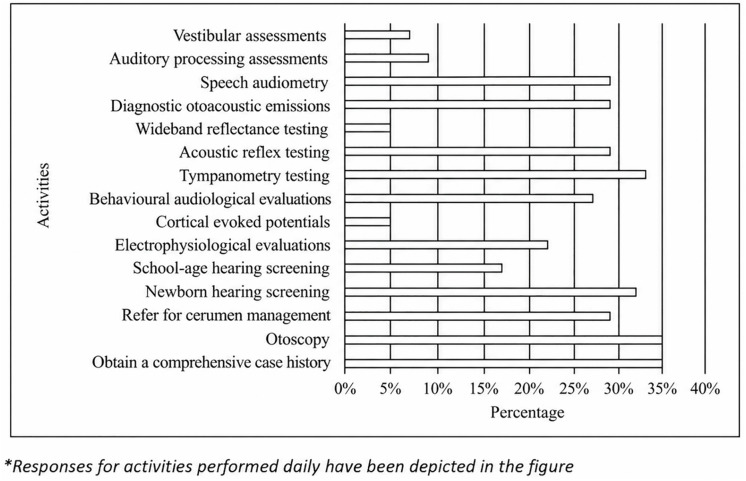
Frequency of performing activities for assessment and diagnosis of auditory and vestibular disorders.

Core pediatric diagnostic procedures were consistently rated as highly important and were frequently performed in clinical practice as depicted in Table 1 and Fig 1. Newborn hearing screening was considered very important by 66.2% of participants, with 35.4% performing it several times daily. Tympanometry was rated very important by 61.5%, and 40% reported performing it several times a day. Case history taking (60%) and otoscopic examination (57.7%) were also routinely conducted, with more than 30% of participants reporting multiple daily use. Behavioural audiological evaluations (50%) and speech audiometry (53.1%) were similarly widely practiced.

**Table 1:** Importance of assessment and diagnosis of auditory and vestibular disorders.

	Response				
Activity	Very Important n (%)	Important n (%)	Moderately Important n (%)	Minimally Important n (%)	Not Important n (%)
Obtain a comprehensive case history	78 (60)	41 (31.5)	10 (7.7)	1 (0.8)	0
Perform otoscopy/video otoscopy and physical examination of the ear	75 (57.7)	45 (34.6)	7 (5.4)	1 (0.8)	2 (1.5%)
Refer for cerumen management	63 (48.5)	41 (31.5)	19 (14.6)	5 (3.8)	2 (1.5)
Perform newborn hearing screening (OAE, AABR)	86 (66.2)	39 (30)	3 (2.3)	2 (1.5)	0
Perform school-age hearing screening (OAE, PTA)	65(50)	51 (39.2)	10 (7.7)	3 (2.3)	1 (0.8)
Perform electrophysiological evaluations (ABR, ASSR)	69 (53.1)	46 (35.4)	12 (9.2)	1 (0.8)	2 (1.5)
Perform cortical evoked potentials (MLR, ALR)	25 (19.2)	44 (33.8)	39 (30)	13 (10)	9 (6.9)
Perform behavioral audiological evaluations	65 (50)	44 (33.8)	17(13.1)	2 (1.5)	2 (1.5)
Perform tympanometry testing	80 (61.5)	42 (32.3)	6 (4.6)	1 (0.8)	1 (0.8)
Perform acoustic reflex testing	67 (51.5)	46 (35.4)	15 (11.5)	1 (0.8)	1 (0.8)
Perform wideband reflectance testing	20 (15.4)	37 (28.5)	47 (36.2)	13 (10)	13 (10)
Perform diagnostic otoacoustic emissions	62 (47.7)	46 (35.4)	16 (12.3)	2 (1.5)	4 (3.1)
Perform speech audiometry (SAT, SRT)	69 (53.1)	43 (33.1)	14 (10.8)	2 (1.5)	2 (1.5)
Perform auditory processing assessments	43 (33.1)	42 (32.3)	33 (25.4)	8 (6.2)	4 (3.1)
Perform vestibular assessments	39 (30)	45 (34.6)	26 (20)	12 (9.2)	8 (6.2)

OAE = Otoacoustic Emissions; AABR = Automated Auditory Brainstem Response; PTA = Pure Tone Audiometry; ABR = Auditory Brainstem Response; ASSR = Auditory Steady State Response; MLR = Middle Latency Response; ALR = Auditory Late Response; SAT = Speech Awareness Threshold; SRT = Speech Reception Threshold.

In contrast, advanced diagnostic procedures demonstrated comparatively lower clinical utilization. Although electrophysiological evaluations were considered very important by 53.1% of participants, only 14.6% reported performing them several times daily. Cortical evoked potentials were rarely incorporated into practice, with 35.4% of audiologists indicating that they did not perform them. Wideband reflectance testing showed the lowest level of integration, with 48.5% never performing the procedure and only 3.1% using it frequently. Similarly, auditory processing assessments were not performed by 33.1% of participants, while vestibular assessments were excluded by 43.1%. Overall, fundamental assessment procedures were routinely implemented, whereas specialized diagnostic tools showed limited adoption in clinical practice.

### Habilitation and rehabilitation practices

**Fig 2:**
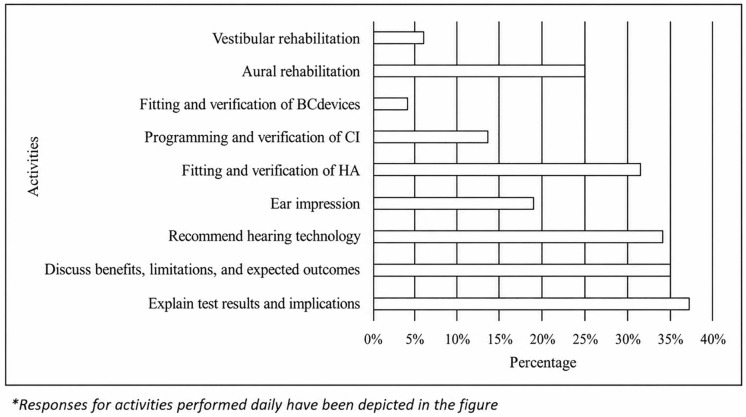
Frequency of performing activities in habilitation and rehabilitation.

The importance and frequency of practicing of different habilitation and rehabilitation practices have been illustrated in Table 2 and Fig 2.

**Table 2:** Importance of activities in habilitation and rehabilitation.

			Response		
Activity	Very Important n (%)	Important n (%)	Moderately Important n (%)	Minimally Important n (%)	Not Important n (%)
Explain test results and implications to the patient/family/caregiver	89 (68.5)	37 (28.5)	4 (3.1)	0	0
Discuss benefits, limitations, and expected outcomes for the hearing device with the patient/family/caregiver	88 (67.7)	35 (26.9)	7 (5.4)	0	0
Recommend hearing technology based on audiometric findings (traditional hearing aids, cochlear implant, bone conduction, remote microphones)	86 (66.2)	37 (28.5)	6 (4.6)	1 (0.8)	0
Perform ear impression	66 (50.8)	44 (33.8)	15 (11.5)	2 (1.5)	3 (2.3)
Perform fitting and verification of hearing aids	84 (64.6)	35 (26.9)	7 (5.4)	2 (1.5)	2 (1.5)
Perform programming and verification of cochlear implants	73 (56.2)	40 (30.8)	9 (6.9)	2 (1.5)	2 (1.5)
Perform fitting and verification of bone conduction devices	51 (39.2)	49 (37.7)	17(13.1)	7 (5.4)	6 (4.6)
Perform aural rehabilitation	74 (56.9)	39 (30.0)	14 (10.8)	2 (1.5)	1 (0.8)
Perform vestibular rehabilitation	53 (40.8)	39 (30.0)	25 (19.2)	8 (6.2)	5 (3.8)

Counselling-related activities emerged as the most emphasized components of habilitation and rehabilitation. Explaining test results was rated very important by 68.5% of participants, with 48.5% performing this activity several times daily. Discussing hearing device options (67.7% very important) and recommending appropriate hearing technology (66.2%) were also frequently practiced, with over 38% performing these activities multiple times each day. In comparison, rehabilitative procedures were less consistently implemented. Hearing aid fitting and verification were rated very important by 64.6% of participants; however, only 20.8% reported performing them several times daily.

Ear impression procedures were most performed weekly (27.7%), while 14.6% did not perform them at all. Cochlear implant programming was excluded from practice by 45.4% of participants, and bone conduction device management was not performed by 57.7%. Although aural rehabilitation was considered very important by 56.9% of participants, 19.2% did not provide this service. Vestibular rehabilitation was excluded by 46.9% of audiologists. These findings indicate that counselling and hearing device recommendations were routinely delivered, whereas advanced rehabilitative services were less commonly practiced.

### Case management and counselling practices

**Fig 3:**
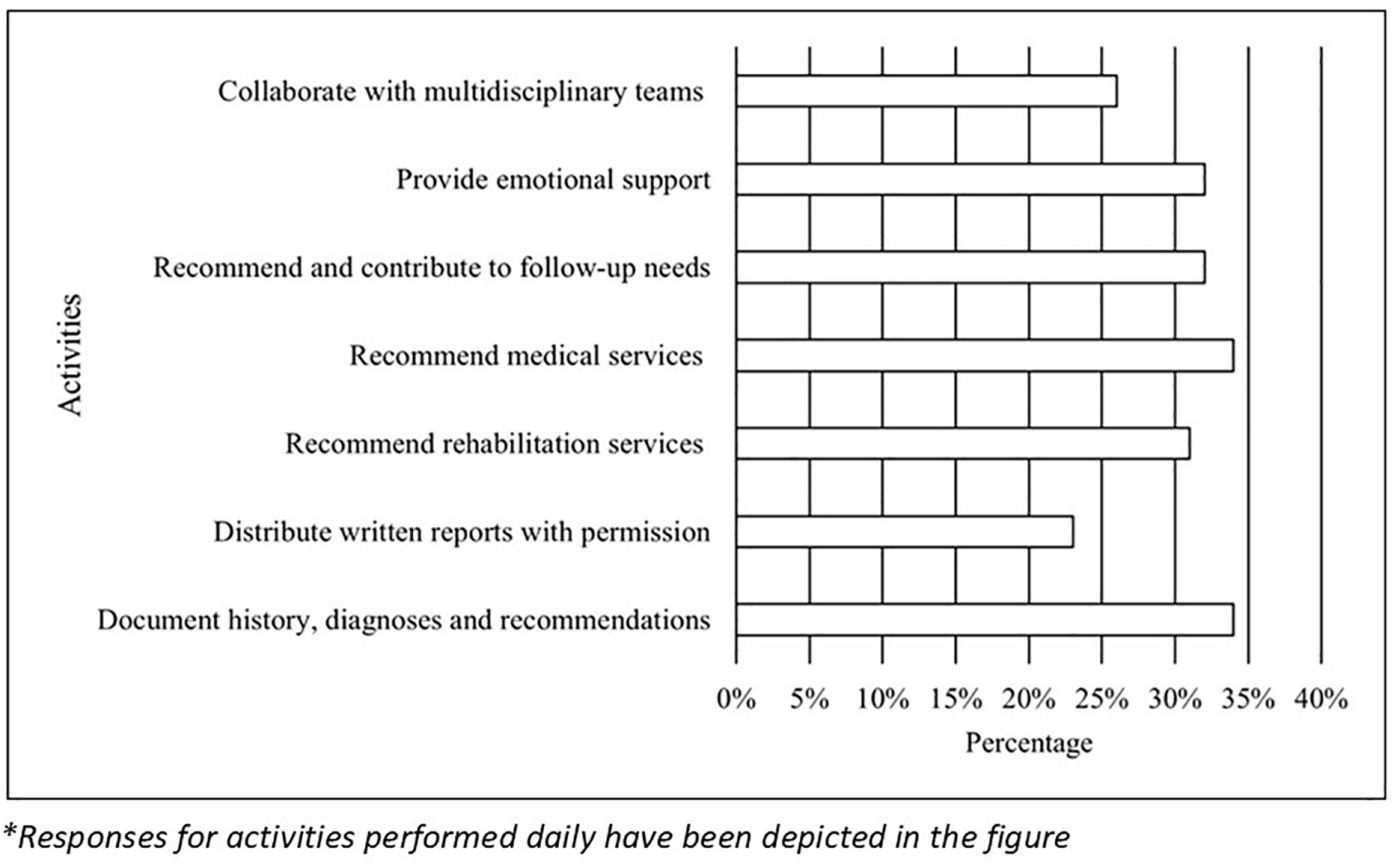
Frequency of activities related to case management and counselling.

The responses for importance and frequency of practices related to case management and counselling are depicted in Table 3 and Fig 3. Case management activities were widely regarded as important and were routinely incorporated into clinical practice. Documentation of clinical findings was rated very important by 66.2% of participants, with 46.9% performing it several times daily. Rehabilitation referrals were considered very important by 63.1%, and 34.6% reported making such referrals several times daily. Similarly, medical referrals were rated very important by 65.4% of participants, with 38.5% carrying them out frequently. Follow-up recommendations were considered very important by 64.6% of participants.

**Table 3:** Importance of activities related to case management and counselling.

			Response		
Activity	Very Important n (%)	Important n (%)	Moderately Important n (%)	Minimally Important n (%)	Not Important n (%)
Document history, diagnoses, and recommendations	86 (66.2)	39 (30.0)	5 (3.8)	0	0
Distribute written reports with permission to the parent/caregiver, referral source, and school	70 (53.8)	40 (30.8)	18 (13.8)	2 (1.5)	0
Recommend rehabilitation services (speech therapy, occupational therapy, physiotherapy)	82 (63.1)	38 (29.2)	10 (7.7)	0	0
Recommend medical services (ENT, developmental paediatrics, mental health)	85 (65.4)	39 (30.0)	6 (4.6)	0	0
Recommend and contribute to audiologic follow-up needs	84 (64.6)	36 (27.7)	10 (7.7)	0	0
Provide emotional support and empathy to the patient/family/care	73 (56.2)	43 (33.1)	13 (10.0)	1 (0.8)	0
Collaborate with multidisciplinary teams regarding the patient’s audiologic needs	78 (60.0)	40 (30.8)	11 (8.5)	1 (0.8)	0

Provision of emotional support was emphasized by 56.2% of audiologists, with more than 30% providing such support several times daily. Collaboration with multidisciplinary teams was regarded as very important by 60% of participants; however, the frequency of collaboration varied, with 30% collaborating several times daily and others reporting less frequent involvement. Overall, the findings demonstrate strong engagement in documentation, referrals, follow-up planning, and family-centred care practices.

### Professional and regulatory practices

**Fig 4:**
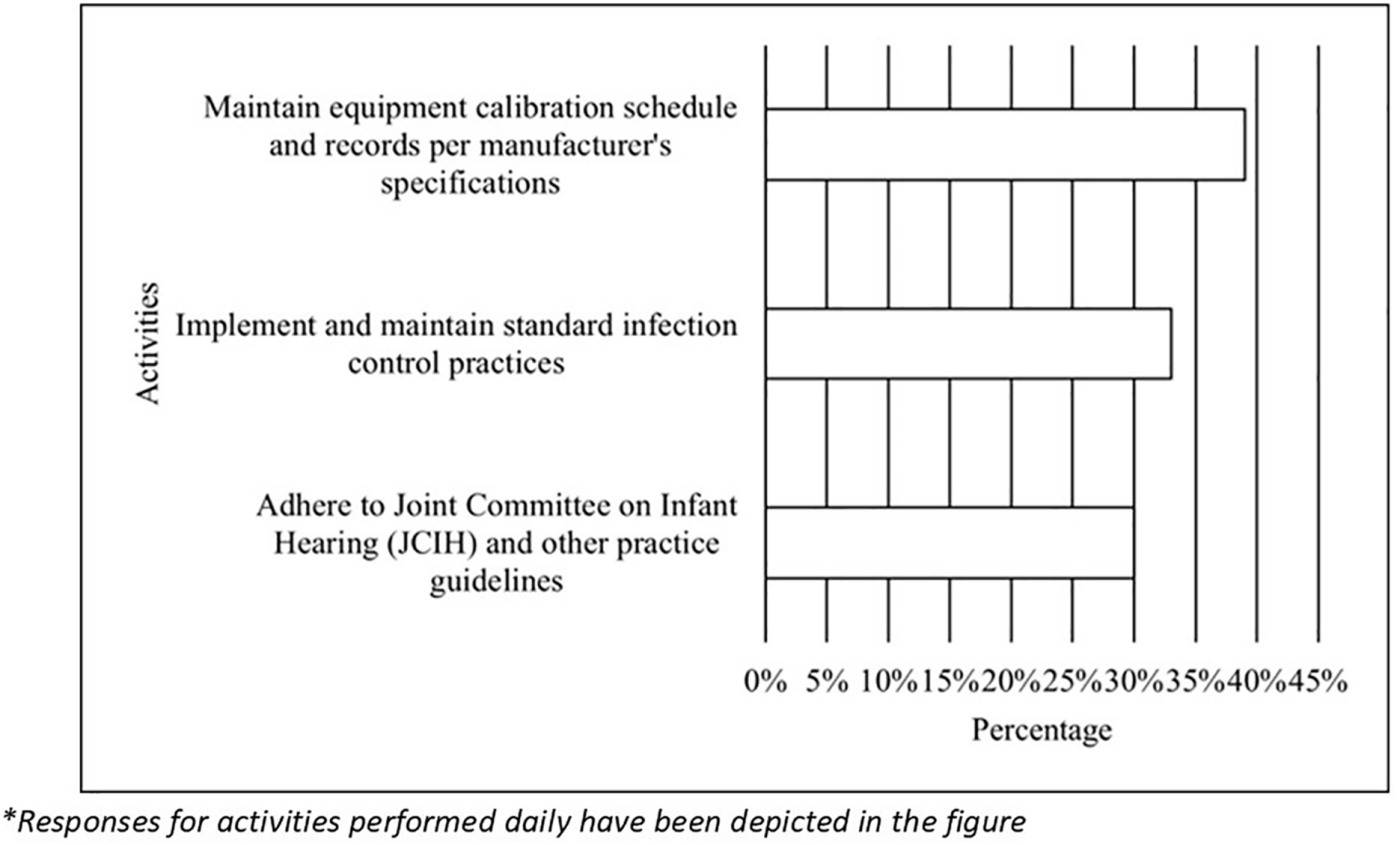
Frequency of activities related to professional and regulatory practices.

Professional and regulatory responsibilities were generally recognized as important aspects of pediatric audiology practice as shown in Table 4 and Fig 4. Equipment calibration was rated very important by 64.6% of participants, followed by infection control practices (56.9%) and adherence to clinical guidelines (55.4%). Regarding implementation frequency, equipment calibration was performed daily by 39.2% of participants and several times daily by 25.4%. Infection control practices were carried out daily by 33.1% and several times daily by 25.4%.

**Table 4:** Importance of professional and regulatory practices.

			Response		
Activity	Very Important n (%)	Important n (%)	Moderately Important n (%)	Minimally Important n (%)	Not Important n (%)
Adhere to Joint Committee on Infant Hearing (JCIH) and other practice guidelines	72 (55.4)	47 (36.2)	10 (7.7)	1 (0.8)	0
Implement and maintain standard infection control practices	74 (56.9)	40 (30.8)	14 (10.8)	1 (0.8)	1 (0.8)
Maintain equipment calibration schedule and records per manufacturer’s specifications	84 (64.6)	37 (28.5)	8 (6.2)	1 (0.8)	0

Adherence to clinical guidelines demonstrated comparatively lower consistency, with only 24.6% of participants reporting implementation several times daily, while 7.7% indicated that they did not follow clinical guidelines in practice.

## Discussion

The present study examined pediatric audiology practices among audiologists in India and identified a predominantly young and early-career workforce (mean age = 28.36 ± 5.43 years; mean experience = 4.92 ± 5.41 years). Clinical experience plays an important role in decision-making, confidence, and the utilization of advanced diagnostic technologies [27]. Younger clinicians tend to have a greater familiarity with current evidence-based guidelines. However, they often face challenges such as limited clinical independence, less experience managing complex pediatric cases, and restricted access to advanced diagnostic equipment [28]. The predominance of female clinicians and urban-centred practice settings observed in the present study is consistent with previous findings in Indian audiology practice [19,29].

More than half of the participants possessed a master’s degree, whereas only a small proportion held doctoral qualifications, suggesting relatively limited research engagement within the workforce. Since evidence-based practice relies on the integration of current research evidence into clinical care [7]. The limited number of doctoral-level professionals may reduce the extent to which advanced and research-driven clinical practices are adopted. Furthermore, only a limited proportion of audiologists reported predominantly pediatric caseloads, while most managed mixed clinical populations. Thus, the findings should be interpreted considering varying levels of pediatric clinical caseloads among the participants. As the study included audiologists with diverse caseloads, the reported frequency of performing specific pediatric procedures may reflect the differences in clinical exposure and scope of practice in addition to resource availability, infrastructure, access to training, budgetary considerations and barriers to implementation. The low reported frequencies may simply reflect the nature of their clinical practice rather than barriers to implementation or underutilization of recommended procedures. Pediatric audiological assessment uses a comprehensive test-battery approach, as no single procedure can independently determine the nature and degree of hearing loss [30–31]. The findings of the present study indicate that Indian audiologists strongly adhere to core pediatric audiology practices, including case history, otoscopic examination, tympanometry, behavioural audiometry, speech audiometry, and newborn hearing screening. Conversely, advanced diagnostic procedures are less frequently employed, likely due to constraints in infrastructure, equipment availability, training, and accessibility.

Case history remains a critical component of pediatric assessment as it guides test selection and interpretation by providing developmental, medical, and familial information. Since young children are unable to self-report symptoms, parental observations play a vital role in identifying risk indicators and guiding follow-up care [32]. Early identification and timely intervention are essential for improving developmental outcomes in children with hearing loss [33]. Similarly, otoscopic examination supports accurate interpretation of audiological findings by identifying outer and middle ear abnormalities, although variability in clinician training and competence has been documented [34–35].

Newborn hearing screening using otoacoustic emissions (OAE) and automated auditory brainstem response (AABR) was widely practiced among participants, indicating adherence to JCIH [32] guidelines. Nevertheless, barriers such as inadequate follow-up systems, workforce shortages, equipment limitations, and inconsistent program implementation continue to affect hearing screening services in India [36–38]. Behavioural audiometry remains the preferred method for evaluating functional hearing and detecting minor degree of hearing loss [39]. Tympanometry and acoustic reflex testing were also widely used, supporting accurate evaluation of middle ear and auditory pathway functioning [40].

Despite the clinical significance of electrophysiological procedures such as ABR and ASSR, were considered clinically important, they were performed less frequently, likely due to the need for specialised equipment, expertise, and infrastructure [32]. Similarly, the clinical uptake of cortical auditory evoked potentials, wideband reflectance testing, auditory processing assessments, and vestibular evaluations was limited. Limited awareness, insufficient training, high equipment costs, and restricted access to advanced facilities may contribute to their infrequent in routine pediatric practice [38–40].

In the field of rehabilitation, there is a prioritisation of counselling and hearing technology recommendations, reflecting the growing emphasis on family-centred pediatric care [41]. However, advanced rehabilitative services, such as hearing-aid verification, cochlear implant programming, bone conduction device management, and structured aural rehabilitation, were less consisten [42]. These findings may reflect disparities in technology access, insufficient specialized training, and the concentration of advanced rehabilitation services in specialized tertiary centers [20,43]. Early intervention remains strongly associated with improved language, communication, and developmental outcomes, emphasizing the need for broader access to comprehensive pediatric rehabilitation services [43].

Patient- and family-centred care is increasingly recognized as an essential component of pediatric audiology practice worldwide, although cultural and contextual factors may influence family participation and decision-making processes [44]. In India, delayed identification of hearing loss and variability in service accessibility further highlight the importance of effective counselling and coordinated management [45]. The findings of the present study reveal that audiologists place significant emphasis on counselling, documentation, referrals, and multidisciplinary collaboration.

Comprehensive documentation was widely valued because it facilitates monitoring of hearing status, amplification outcomes, and developmental progress while supporting clinical decision-making, interdisciplinary communication, and medico-legal requirements. Referral practices were also commonly reported, reflecting the multidisciplinary nature of pediatric hearing healthcare. The JCIH [31] emphasizes coordinated care within Early Hearing Detection and Intervention (EHDI) systems to ensure continuity from screening through intervention and long-term follow-up. Frequent follow-up recommendations observed in the present study align with these principles.

Participants emphasized the significance of multidisciplinary collaboration, which supports family-centred intervention models in pediatric hearing care. Emotional and psychosocial counselling was also recognized as a valuable component of service delivery, as hearing loss affects not only the child but also family adaptation and psychosocial well-being [46]. However, variability in counselling practices suggests a continued need for structured training programs and counselling-focused professional development initiatives [21].

In India, the roles and responsibilities of speech and hearing professionals differ based on educational qualifications and clinical training [47]. Ethical and professional competence, including adherence to regulatory guidelines, is essential for ensuring safe and effective clinical practice [48]. The present findings reveal that Indian audiologists acknowledge the importance of professional responsibilities such as infection control, equipment calibration, and adherence to evidence-based guidelines.

Evidence-based practice requires clinicians to integrate research evidence, clinical expertise, and patient needs into decision-making [7]. Regular equipment calibration is particularly important because calibration errors may compromise diagnostic accuracy and potentially affect a child’s developmental outcomes. Existing guidelines emphasize the importance of standardized protocols and periodic calibration checks to maintain quality assurance in audiological practice [49]. Although participants recognized the importance of clinical guidelines, variations in adherence highlight the need for stronger implementation strategies, continuing professional education, and monitoring systems to promote evidence-based pediatric audiology services in India.

### Implications for practice

The findings of the present study indicate that routine pediatric audiology practices, including newborn hearing screening, behavioural assessment, counselling, and case management, are well established among audiologists in India. However, advanced diagnostic procedures and specialized rehabilitation services were reported less frequently. These findings highlight opportunities to further strengthen pediatric audiology practice through continuing professional education, improved access to specialized diagnostic and rehabilitative technologies, and greater implementation of evidence-based clinical guidelines. Strengthening multidisciplinary collaboration and promoting standardized pediatric care pathways may also support the delivery of comprehensive hearing healthcare services for children.

### Limitations

The present study was based on self-reported online survey responses and may not fully reflect actual clinical practices, particularly in rural or low-resource settings. Direct observation of clinical procedures and verification of equipment were not performed, limiting confirmation of reported practices. The study focused primarily on the frequency of procedures and did not assess factors such as clinician competency, supervision, infrastructure, or availability of specialized training. As a cross-sectional study, it could not evaluate longitudinal changes or causal relationships, and child developmental outcomes were not examined.

### Future directions

Future research should investigate barriers to implementing advanced pediatric audiology practices in India. Greater emphasis is needed on hands-on training, mentorship, and continuing professional education in pediatric assessment, electrophysiological testing, hearing-aid verification, and rehabilitation. Improving access to child-specific diagnostic and rehabilitative equipment across clinical settings is equally important. The development of standardized national guidelines and structured care pathways from screening to intervention and follow-up may further strengthen pediatric audiology services. Enhanced multidisciplinary collaboration and family-centred approaches are also essential for improving long-term outcomes in children with hearing loss.

## Conclusion

The present study demonstrates that pediatric audiology services in India are well established with respect to routine clinical practices such as newborn hearing screening, behavioural assessment, counselling, and case management. However, advanced diagnostic procedures and specialized rehabilitative services remain underutilized despite being recognized as clinically important. These findings highlight the need for improved infrastructure, greater access to advanced technologies, specialized professional training, and stronger implementation of evidence-based guidelines. Strengthening these areas may help reduce variability in pediatric audiology practices and enhance the quality of hearing healthcare services for children in India.
